# Medical News and Items

**Published:** 1877-12

**Authors:** 


### Medical News and Items.

The Illinois State Board of Health has issued the following circular which will explain itself.

Chicago, Nov. 10, 1877.

To the Medical Profession of Illinois:

To facilitate the obtaining of the Certificates for Practice issued by this Board, and the registration of births and deaths, required by State law, the following information is furnished:

All persons (whether male or female) practicing medicine, in any of its departments, in this State, are divided into two classes. First, practitioners who have been practicing medicine ten or more years, within this State. Second, those who have been practicing medicine in this State for a period of time less than ten years, from July 1, 1877.

This second class is divisible into two sections: First, those who can furnish satisfactory proof of having received diplomas as graduates in medicine; or licenses, from some legally chartered medical institution in *good standing*.

Second, those who are practicing medicine in any of its departments, who have neither a diploma or license, (and who are required by law to undergo examination by the State Board, for its certificate entitling them to practice medicine and surgery in the State of Illinois.)

Section four of the State Board of Health Act, requires that all physicians and midwives, without any exception, practicing in this State, shall register their names and postoffice address with the County Clerk of the county where they reside. This is now being enforced, and the signatures of all are required.

Physicians are likewise required to have recorded in the office of the County Clerk, the Certificate which may be received from the State Board of Health; and, in case of removal to another county in the State, to have the certificate recorded in that county also.

In case a physician practices in another county, or counties, in this State, adjoining the one in which he lives—he having recorded his Certificate in the county in which he lives—it will not be required of him to place his Certificate upon record in the other county or counties; but he must register his name, also, in the county or counties in which his practice extends, and make his returns of births and deaths occurring in those counties to the respective County Clerks. No such registration will be required in cases of professional consultation with resident physicians.

Physicians residents of adjoining States, who practice in this State in counties adjoining the one in which they reside, are required to take out Certificates of Practice, as required of residents of this State, and record the same in the office of County Clerk of the county or counties in which their practice extends, and to make returns of births and deaths in those counties to the County Clerk, and in every respect conform to all the requirements of the law upon residents of this State.

Certificates will, when desired, be issued to non-graduates of ten or more years practice in this State, on their affidavit of such practice, supported by evidence of good standing in a medical society, or the recommendations of reputable professional or other men, who have known them during their term of practice.

These Certificates are different from those granted to graduates, and are issued at the discretion of the Board.

Applications for Certificates, or for blank affidavits, or for appointments for examinations, may be made to the President, Dr. J. H. Rauch, 202 State street, Chicago. Diplomas, or licenses, and affidavits properly filled out and acknowledged, together with the fees for certificate, may be sent to the same office.

For convenience, diplomas, licenses, affidavits and fees may be sent from their immediate neighborhood to H. Wardner, M. D., Cairo; W. M. Chambers, M. D., Charleston; J. M. Gregory, LL. D., Champaign; N. Bateman, LL. D., Galesburg; and A. L. Clark, M. D., Elgin.

A special form of blank affidavit has been prepared for those who have lost their diplomas or licenses by fire or otherwise, and may be had upon application.

It is not required that the ten years of practice in this State shall be consecutive years; but the sum total of practice in this State must amount to ten years, from and before July 1, 1877. No allowance is made for the time in practice in any other State, or service in the army.

Certificates of graduation will be issued to graduates who have practiced medicine ten or more years in this State, upon their making an affidavit to that effect, at the same time giving name of College, place and date of graduation, without their being required to send diploma to the Board for verification, by paying the usual fee. It is not obligatory upon this class of practitioners to take out a Certificate.

In view of possible legal questions in practice, it will, no doubt, be better for all who can obtain Certificates to do so.

Owing to the fact that many of those who have been in practice over ten years supposed that nothing at all was required of them, the Board addressed a communication to Hon. J. K. Edsall, Attorney General of the State, asking what construction the Board should put upon this part of the Act, and their duty. His reply was as follows:

"The proviso in question is in these words: '*Provided*, that the provisions of the Act shall not apply to those who have been practicing medicine ten years within the State.' I have no question that the effect of this proviso is to exempt all persons who fall within its terms, from the penalties imposed by the Act in question. But, considering the entire scope of the Act, I am of the opinion that the duty is incumbent upon your Board to ascertain, as far as practicable, the names of all those who are entitled to the benefits of that proviso; and that it is the duty of those who have thus practiced medicine ten years within the State to furnish you, on request, evidence of the fact by affidavit, or otherwise. Your Board may, with propriety, issue in return some proper certificate, which will show their right to practice under that proviso of the law."

*All* therefore who claim exemption from the penalties of the Act, in consequence of the fact of their having practiced medicine in this State for ten years previous to July, 1, 1877, and who have not procured Certificates, will be called upon by the Board, after December 31, 1877, to make affidavit to that effect, and file the same with the Board, thus establishing their status legally.

In regard to practitioners of medicine who have been practicing in this State less than ten years, the law requires that those who are graduates in medicine, or who have licenses from legally chartered medical institutions in good standing, must actually present, for verification, to the State Board, their diplomas or licenses, and, in addition, such other satisfactory proofs as may be necessary. Affidavit must be made that the person presenting the diploma, or license, is the lawful possessor of the same. Graduates may present their diplomas or licenses, and affidavits by letter or proxy. The affidavit should mention date and place of graduation, and name of medical college; length of practice in this State, as well as present place of residence.

The fee for certificates of graduation is fixed by law at one dollar.

Certificates of Graduation must be recorded in the office of the County Clerk. In fact, it is better that all Certificates issued by the Board should be so recorded.

It is required by law that all persons practicing medicine less than ten years in any of its departments, who are not graduates in medicine or licentiates, shall be examined directly by the Board, and then Certificates will be issued to those passing the examination. The Certificate is to be recorded in the office of the County Clerk. Candidates for examination are required to pay a fee of five dollars, in advance—to be returned to them if the Certificate be refused.

Examinations may be, in whole or in part, in writing, or orally; and shall be of an elementary and practical character, but sufficiently strict to test the qualifications of the candidate as a practitioner.

Meetings of the Board, for the examinations of candidates for Certificates of practice, and for the verification of diplomas and licenses, and taking of affidavits, are appointed for the following dates and places:

GALESBURG—Thursday, December 6th, beginning at 10 A. M., at the Union Hotel.

CHAMPAIGN.—Thursday, December 20th, beginning at 10 A. M., in the parlors of the Industrial University.

SPRINGFIELD.—Thursday, January 10th, 1878, at 10 A. M., at the State House (the regular annual meeting).

Meetings for examination will also be held at Charleston and Chicago, in January, and other points, the location to be determined by the proximity of applicants, as the Board does not wish to cause any unnecessary loss of time to candidates for examination. The dates of these meetings will be duly announced.

Practitioners who are required by law to be examined—namely, those who have neither a diploma or license, and have practiced medicine in this State less than ten years, must present themselves in person before the Board. No examination papers can be sent out to individuals; and no examination can be limited to any one or two special topics, or branches of study or practice. The examinations will be conducted by the entire Board, and upon all branches usually taught in medical schools.

Questions relating to special methods or forms of practice, or therapeutics, will be referred for examination to the various individual members of the Board, as may be indicated.

A proper and reasonable regard and respect will be paid to the personal and peculiar views of practice, if any such there should happen to be, of the candidates presenting themselves for examination.

Candidates for examination must fill out a blank form, which may be obtained on application. This must be sent to the office of the Board, and, if approved, they will be notified to appear for examination.

The forms pertaining to the State system of registration of births, deaths, and marriages, are now prepared, and on and after December 1st, registration will be enforced. It is to be hoped that there will be a prompt and cheerful compliance with this, one of the most important sanitary and civil measures ever inaugurated in this State.

Much time and correspondence may be saved by applicants stating their cases clearly and succinctly.

No certificate will be issued until the fee of one dollar is paid in advance. It will also be remembered that the finances of the Board are exceedingly limited, and correspondents will please see that the postage is fully prepaid on their communications. It is much better that diplomas sent to the Board for verification be forwarded by express than by mail. The office of the Board, for the present, is at Chicago.

The hearty coöperation of all medical practitioners in good standing is expected. It is not alone a duty they owe to the people, but a matter of interest to themselves, as it is the determination of the Board to protect and advance the legitimate practitioner.

"College of Physicians and Surgeons, City of Keokuk, Iowa."—The leading announcement of the medical schools of this country is issued by the college bearing the above name. Its announcement "leads" in several particulars. It "leads," for instance, in pretentiousness, it "leads" in magnificence of proof-errors, it "leads" in extreme economy of expense incurred in its production, it "leads" in the horrible taste displayed in a three-inch cut, most diabolically executed, affording a "view of one of the college dissecting rooms." The cut represents an apartment "over 50 feet in length," containing eight tables, an old pewter injecting syringe of the pattern used sixty-five years ago, a student at a table, a demonstrator, presumably on foot, "attitudinizing" for a photograph, a janitor, a blown-up, shriveled gut on a gas-pipe, eight cadavers in various stages of disappearance, one of them in a position for perineal dissection, with a urethral sound *in situ*, two skulls, a dead baby, and an infinitude of cracks in the floor. The announcement "leads" in other particulars: in general information, for example, concerning the city of Keokuk itself. We are informed that Keokuk is "one of the most beautiful cities in the Mississippi Valley;" that "it is the location of the great ship canal around the Des Moines rapids of the Mississippi, which has been formally opened" (evidently the canal is here meant), "and has cost our government over four millions of dollars,"—both of which items of general information are of incalculable value to the student attending lectures in the College of Physicians and Surgeons, City of Keokuk, Iowa. The announcement "leads" also in the variety of text-books recommended, which "may be purchased at reasonable prices at the extensive bookstores of our city"—(prices are, of course, not "reasonable" at "the bookstores of our city", which are not "extensive"). Here are recommended, *e. g.*, "*Cruolhier's*" Anatomy, "*Kliss*'" Physiology, "*Bartholind's*" Materia Medica, "*Cazeau's*" Obstetrics, "*Deon's*" Medical Jurisprudence, "*Rockatansky's*" Pathological Anatomy, and other books too numerous to mention.

The particular, however, in which this document "leads" is in the announcement of the virtues of the college in question, which announcement is without parallel in this or in any other country of the globe, in the stupendousness of its disregard for the ways of good grammar. Copies of this announcement should be forwarded at once to the various university historical libraries of the country, not forgetting that of the Surgeon General's office, for preservation and future reference.

After announcing in hackneyed terms "the continued prosperity and success of the Institution," we are treated to this grammatical effusion :

"No words of ours can be half so convincing to the physician or medical student, who wishes to attend a course of medical lectures, of the usefulness and success of the Institution, than by reference to our one thousand Alumni, whose names are herewith presented, and whose medical reputation and influence is felt throughout the entire Northwest."

We are informed that the faculty at Keokuk can "look off and lecture without notes;" that they deliver "*extemporaneous lectures*," and this is why such lectures are an advantage : "Years of experience has proved the great advantage of *extemporaneous* lectures over those *written and read* before the medical student." We are also informed "that the College of Physicians and Surgeons offer greater inducements to the student, and at less cost, than any other regular Institution in the country." We learn here also that an "*Ad Euendum* Degree" is conferred when occasion requires.

Enough is as good as a feast. More quotations from this remarkable announcement might discourage the publication of others. We trust that no one will impudently suggest that the Trustees of the "College of Physicians and Surgeons, City of Keokuk, Iowa," add a Professor of Grammar and Rhetoric to their Faculty.

WALSH'S PHYSICIANS' CALL-BOOK, AND HANDY LEDGER.—The *Call Book* has reached its third edition. The astonishing rapidity with which it has become a great favorite with the profession is the best proof of its merits. The *Handy Ledger* is another proof of Dr. Walsh's knowledge of the practical needs of the profession. Physicians, as a rule, are not very good business men nor have they the time requisite to keep their account books in a business-like manner. The *Handy Ledger* now offers a system of book-keeping so simple, yet complete, that the busiest physician needs but a few minutes per day to keep his accounts posted up.

The call-book costs $1.50; the ledger, $2.50; both can be obtained of R. Walsh, M. D., 326 C street, Washington, D. C.

The Illinois State Medical Register for 1877–8 is now ready, and can be obtained for one dollar and fifty cents by applying to Mr. W. T. Keener, 63 and 65 Washington street, Chicago.

The *Chicago Medico-Historical Society*, which has hitherto published an annual directory of Chicago physicians, has enlarged the scope of its work, and with the coöperation of the Illinois State Medical Society, issued a register of regular physicians in the entire State. The publication committee does not claim that the list comes any where near perfection, as in a first attempt at compiling such a list "the omission of worthy names and the admission of unworthy ones, are defects always unavoidable." The committee, therefore, solicits criticism and suggestions, by which the next edition could be improved.

Besides the list of physicians, the volume contains a great deal of valuable information; *e. g.*, Code of Ethics of the American Medical Association; Constitution and By-laws of the Illinois State Medical Society; complete list of the National, State, and County Medical Societies; A Directory of the Charitable and Benevolent Institutions, Hospitals and Dispensaries in Illinois ; and a Digest of the Laws of the State of Illinois, and of the Ordinances and regulations of the City of Chicago, relating to public health and interesting the medical profession.

### TO OUR SUBSCRIBERS.

With the conclusion of this volume expires the period of subscription of most of the names on our list. We shall be under obligations to those of our friends who will promptly remit to us their favors, either for the entire year or for the volume which will be concluded with the number of June, 1878.

We beg leave to call attention to the necessity of prompt remittances, as we can thus estimate for the future in such a way as to greatly improve the character of the JOURNAL.

